# Reviews and Book Notices

**Published:** 1878-07

**Authors:** 


### Reviews and Book Notices.

Physics of the Infectious Diseases. By C. A. Logan, A. M., M. D. Published by Jansen, McClurg & Co., 1878.

We commenced the "interview" of this little book with an amount of partiality, as it is a child of Chicago, but were soon greatly disappointed to find that it did not do justice to the place of its birth. The title is novel, and we expected a treat; perhaps that is one reason why we feel the disappointment the more acutely. The style of medical literature may by some be thought to be of secondary importance, but if it is so, the secondary position is in close proximity to the primary. In the present instance the style is heavy and verbose. For instance, at the bottom of page 48, on the subject of ozone we read: "The whole subject is one of immense difficulty, as involving forces aud agencies of nature with which we are but little acquainted, and with whose ethereal existence we have but few channels of communication." What additional force or expression does the last sentence, "and with whose ethereal," etc., give to the already fully stated idea? Again, in the section commencing on page 46, our attention is called to the effect of ozone on "man and animals." Whatever view the author may take of the relation of man to the lower animals, man must be admitted as part of the animal world. The same remark will apply to the heading of the next section: "Its effects upon *insects and aerial organisms.*" On page 43 the author says, "most unfortunately, the reported result of all these observations," relative to ozone, "are so very conflicting as to furnish no data *upon which to erect any certain lights which may go to illuminate the dark corridors of disease-causation.*" The italics are ours, but we would suggest that we do not erect lights on data; and certainly the "*disease-causation*" should scarcely be likened to a house with dark corridors. We had noted down numerous other instances still more glowing than these, but we cannot encroach more on this point. One more phrase, however, to substantiate our charge of verbosity. The author suggests an amendment to the time honored Latin dictum, *mens sana*, etc., which he would improve thus: "A sound nervous system makes a sound body." In terse English: Sound centres, sound whole.

However we may differ in opinion as to the value of style in medical literature, there will not be two opinions as to the right of accuracy to sway the sceptre. And here our author lays himself open to severe assaults. On page 143 we read: "Though the chemist by analysis may tell us the constituent atoms of certain products of the living being, and though he may be able even to reconstruct or produce some simple forms in his laboratory, yet his unimportant results in this direction only prove the existence of a force presiding over the methods of vital chemistry, which render him impotent to construct such eminently vital products as urea.

Now, in the first place, the chemist can and has for some years past manufactured *urea*. Moreover, it ill becomes any one who shows himself so little acquainted with what chemisty has done, even though he may be the most prejudiced opponent of the enthusiastic chemist, to dub his work as unimportant results, in face of the remarkable achievements which have been made in physiological chemistry in the last ten years.

In the section on nervous bankrupts, the trite observation is brought before us, page 196, "A man may labor hard at some merely mechanical work, and not suffer a tithe of that exhaustion which follows a much shorter period of brain effort by the same individual." That constitutes the substance of the whole section on "Nervous Bankrupts," and even in this observation we are obliged to differ from our author. It is not work that wearies a man, it is *worry*. It is not the work that we do that affects the nervous bankrupts, it is the work that we *fail to do*, having tried. And our opinion is that muscular work tires a man just as much as simple brain work.

There are many points of interesting discussion suggested in the book, but they would take up more space than could be afforded.

